
JOURNAL INFORMATION
==============================
NLM Title Abbreviation: J Mol Cell Biol
Journal ID: J Mol Cell Biol
NLM Journal ID: 101503669
Journal ID: jmcb

Journal of Molecular Cell Biology

ISSN: 1674-2788
EISSN: 1759-4685

ARTICLE INFORMATION
==============================
PMCID: PMC7928739
PMID: 33453103
DOI: 10.1093/jmcb/mjab006
Article ID: mjab006
Article version: 1
Subjects: Highlight, AcademicSubjects/SCI01180

Kidney injury molecule-1: a novel entry factor for SARS-CoV-2

Wan Cheng
Zhang Chun
Department of Nephrology, Union Hospital, Tongji Medical College, Huazhong University of Science and Technology , Wuhan 430022, China
Correspondence to: Chun Zhang, E-mail: drzhangchun@hust.edu.cn
Electronic publication date: 2021 Jan 16
Electronic Location ID: mjab006
Received 2020 Nov 22; Accepted 2020 Dec 29
Copyright: © The Author(s) 2021. Published by Oxford University Press on behalf of Journal of Molecular Cell Biology, IBCB, SIBS, CAS.
Copyright year: 2021
License: This is an Open Access article distributed under the terms of the Creative Commons Attribution License (http://creativecommons.org/licenses/by/4.0/), which permits unrestricted reuse, distribution, and reproduction in any medium, provided the original work is properly cited.
License URL: https://creativecommons.org/licenses/by/4.0/

Page count: 4
edited-state: accepted-manuscript
article-lifecycle: PAP

==============================
### Highlight

### Kidney injury molecule-1: a novel entry factor for SARS-CoV-2

Cheng Wan and Chun Zhang*

Department of Nephrology, Union Hospital, Tongji Medical College, Huazhong University of Science and Technology, Wuhan 430022, China

* Correspondence to: Chun Zhang, E-mail: drzhangchun@hust.edu.cn

Renal involvement occurs frequently among patients with coronavirus disease 2019 (COVID-19) caused by severe acute respiratory syndrome coronavirus 2 (SARS-CoV-2), and acute kidney injury (AKI) is associated with poor prognosis and mortality of COVID-19. The potential mechanisms of AKI include direct virulence of SARS-CoV-2 and indirect mechanisms such as hyperinflammation, hypercoagulation, and unspecific kidney injury factors (Gabarre et al., 2020). The existence of infective SARS-CoV-2 in kidney has been verified providing a definite evidence of renal tropism and direct invasion of SARS-CoV-2 (Braun et al., 2020). Ascertaining the mechanism of SARS-CoV-2 entry will pave the way for preventing the infection and even tackling this epidemic.

The first step for viral invasion is the binding between SARS-CoV-2 spike protein and host cell receptors. Angiotensin-converting enzyme 2 (ACE2) is the first well-confirmed receptor for SARS-CoV-2 (Shang et al., 2020). Receptor-binding domain (RBD) in spike protein directly binds to ACE2, thus promoting the virus–cell fusion and subsequent entry. CD147 is verified as another receptor for SARS-CoV-2 mediating viral entry by endocytosis (Wang et al., 2020). The redistribution of CD147 in tubular epithelial cells from basolateral pattern to circumferential pattern likely facilitates the putative invasion of SARS-CoV-2 from the lumen (Su et al., 2020). Neuropilin-1 highly expressed in the respiratory and olfactory epithelium is also an additional host cell receptor for SARS-CoV-2, and blocking the interaction between neuropilin-1 and viral spike protein reduces SARS-CoV-2 entry (Daly et al., 2020). Besides the above, Yang et al. (2021) recently proposed kidney injury molecule-1 (KIM-1) as a new host factor.

KIM-1 is well-known as a sensitive biomarker of AKI and tubular injury in other kidney diseases. It is not detectable in normal kidney, but markedly upregulated in proximal tubular cells upon injury. In addition, KIM-1 is a double-edged sword in the process of kidney healing and injury. On the one hand, it functions as a scavenger receptor facilitating the clearance of apoptotic and

© The Author(s) 2021. Published by Oxford University Press on behalf of Journal of Molecular Cell Biology, IBCB, SIBS, CAS. This is an Open Access article distributed under the terms of the Creative Commons Attribution License (http://creativecommons.org/licenses/by/4.0/), which permits unrestricted reuse, distribution, and reproduction in any medium, provided the original work is properly cited.

necrotic cells in tubular lumen and participates in the regeneration of injured tubules (Ichimura et al., 2008). On the other hand, chronic KIM-1 expression contributes to tubulointerstitial fibrosis and inflammation (Humphreys et al., 2013). Moreover, KIM-1 is emerging as a critical entry factor for several enveloped viruses, such as Ebola virus and Dengue virus, through direct interaction between phosphatidylserine (PtdSer) binding residues within extracellular immunoglobulin variable (IgV)-like domain and PtdSer on the viral envelope, promoting the subsequent virus internalization (Moller-Tank et al., 2014).

SARS-CoV-2 belongs to enveloped viruses as well. In a recent study by Yang et al. (2021), the role of KIM-1 in SARS-CoV-2 infection has been investigated. It is proposed that KIM-1 is not only a biomarker for SARS-CoV-2-associated AKI, but also a novel receptor that binds to SARS-CoV-2 RBD via IgV domain. Similar attachment has been revealed in SARS-CoV and MERS-CoV, confirming the role of KIM-1 in coronavirus invasion. It has been suggested that ACE2 and KIM-1 co-mediate the viral invasion leading to acute tubular injury, and the subsequent upregulation of KIM-1 further promotes SARS-CoV-2 entry, thus forming a vicious cycle in the kidney. However, how KIM-1 facilitates SARS-CoV-2 internalization after binding remains to be further explored.

Another remarkable finding by Yang et al. (2021) is the designed KIM-1-derived antagonist peptide covering the two SARS-CoV-2-contacting motifs (motif 1: Leu54, Phe55, Gln58; motif 2: Trp112, Phe113) with three glycine as the flexible linker. The peptide successfully blocks the attachment between KIM-1 and SARS-CoV-2, which may help new drug discovery for SARS-CoV-2 infection (Yang et al., 2021).

In summary, Yang et al. (2021) reveals a novel virus entry route for SARS-CoV-2, KIM-1, which also acts as a biomarker for SARS-CoV-2-associated AKI. The finding of new host cell factors may provide promising targets for developing specific and effective interventions against COVID-19.

### References

Braun, F., Lütgehetmann, M., Pfefferle, S., et al. (2020). SARS-CoV-2 renal tropism associates with acute kidney injury. The Lancet *396*, 597-598.

Daly, J. L., Simonetti, B., Klein, K., et al. (2020). Neuropilin-1 is a host factor for SARS-CoV-2 infection. Science *370*, 861–865.

Gabarre, P., Dumas, G., Dupont, T., et al. (2020). Acute kidney injury in critically ill patients with COVID-19. Intensive care medicine, *46*, 1339–1348.

Humphreys, B. D., Xu, F., Sabbisetti, V., et al. (2013). Chronic epithelial kidney injury molecule-1 expression causes murine kidney fibrosis. The Journal of clinical investigation, *123*, 4023–4035.

Ichimura, T., Asseldonk, E. J., Humphreys, B. D., et al. (2008). Kidney injury molecule-1 is a phosphatidylserine receptor that confers a phagocytic phenotype on epithelial cells. The Journal of clinical investigation, *118*, 1657–1668.

Moller-Tank, S., Albritton, L. M., Rennert, P. D., et al. (2014). Characterizing functional domains for TIM-mediated enveloped virus entry. Journal of virology, *88*, 6702–6713.

Shang, J., Ye, G., Shi, K., et al. (2020). Structural basis of receptor recognition by SARS-CoV-2. Nature, *581,* 221–224.

Su, H., Wan, C., Wang, Z. D., et al. (2020). Expression of CD147 and Cyclophilin A in Kidneys of Patients with COVID-19. Clinical journal of the American Society of Nephrology, doi: 10.2215/CJN.09440620.

Wang, K., Chen, W., Zhang, Z., et al. (2020). CD147–spike protein is a novel route for SARS-CoV-2 infection to host cells. Signal transduction and targeted therapy, *5*, 283.

Yang, C., Zhang Y., Zeng X., et al. (2021). Kidney injury molecule-1 is a potential receptor for SARS-CoV-2. Journal of Molecular Cell Biology, doi: 10.1093/jmcb/mjab003.

### Figure Legend

**Figure 1** The dual role of KIM-1 in SARS-CoV-2 infection: biomarker for AKI and entry factor.

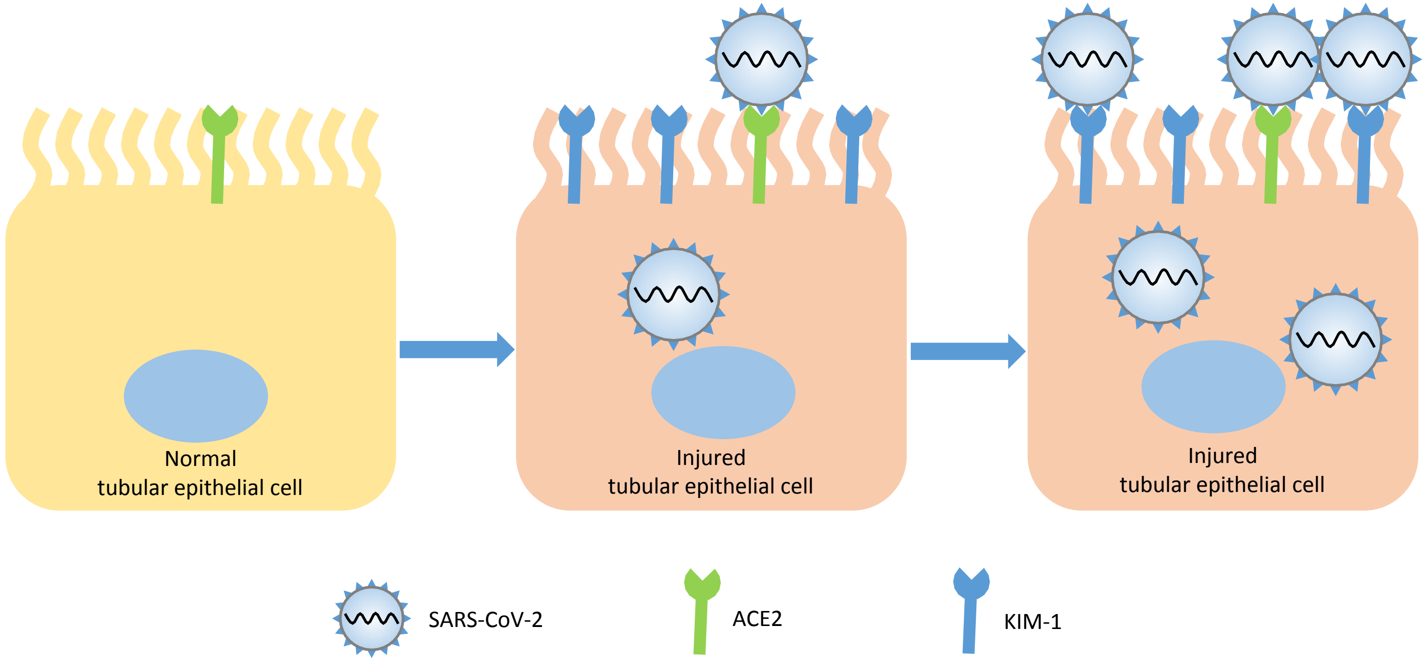

Normal
tubular epithelial cell
Injured
tubular epithelial cell
Injured
tubular epithelial cell
SARS-CoV-2
ACE2
KIM-1
